# Risk factors and survival prediction of pancreatic cancer with lung metastases: A population-based study

**DOI:** 10.3389/fonc.2022.952531

**Published:** 2022-09-21

**Authors:** Zong-Xi Yao, Jun-Hao Tu, Bin Zhou, Yang Huang, Yu-Lin Liu, Xiao-Feng Xue

**Affiliations:** ^1^ Department of General Surgery, Suzhou Wuzhong People’s Hospital, Suzhou, China; ^2^ Department of General Surgery, The First Affiliated Hospital of Soochow University, Suzhou, China

**Keywords:** pancreatic cancer, lung metastases, SEER database, nomogram, decision curve analysis

## Abstract

**Background:**

The risk and prognosis of pancreatic cancer with lung metastasis (PCLM) are not well-defined. Thus, this study aimed to identify the risk and prognostic factors for these patients, and establish predictive nomogram models.

**Methods:**

Patients diagnosed with PCLM between 2010 and 2016 were identified from the Surveillance, Epidemiology, and End Results (SEER) database. Independent risk factors and prognostic factors were identified using logistic regression and Cox regression analyses. Nomograms were constructed to predict the risk and survival of PCLM, and the area under the curve (AUC), C-index, and calibration curve were used to determine the predictive accuracy and discriminability of the established nomogram, while the decision curve analysis was used to confirm the clinical effectiveness.

**Results:**

A total of 11287 cases with complete information were included; 601 (5.3%) patients with PC had lung metastases. Multivariable logistic analysis demonstrated that primary site, histological subtype, and brain, bone, and liver metastases were independent risk factors for lung metastases. We constructed a risk prediction nomogram model for the development of lung metastases among PC patients. The c-index of the established diagnostic nomogram was 0.786 (95%CI 0.726-0.846). Multivariable Cox regression analysis demonstrated that primary site, liver metastases, surgery, and chemotherapy were independent prognostic factors for both overall survival (OS) and cancer-specific survival (CSS), while bone metastases were independent prognostic factors for CSS. The C-indices for the OS and CSS prediction nomograms were 0.76 (95% CI 0.74-0.78) and 0.76 (95% CI 0.74-0.78), respectively. Based on the AUC of the receiver operating characteristic (ROC) analysis, calibration plots, and decision curve analysis (DCA), we concluded that the risk and prognosis model of PCBM exhibits excellent performance.

**Conclusions:**

The present study identified the risk and prognostic factors of PCLM and further established nomograms, which can help clinicians effectively identify high-risk patients and predict their clinical outcomes.

## Introduction

Pancreatic cancer (PC) is the seventh leading cause of cancer-related mortality worldwide, with lethality caused by late presentation at initial diagnosis and a poor response to traditional therapy (1). As a silent cancer, PC is generally asymptomatic at an early stage and is often diagnosed when it progresses to an advanced stage with either locally advanced, unresectable, or metastatic disease. Only 20-30% of patients with pancreatic cancer have a chance to undergo surgical resection at diagnosis. However, approximately 80% of surgically resected pancreatic cancers recur within five years of resection, and more than 60% of patients develop recurrence within two years (2). Thus, PC exhibits high mortality and poor survival, and only 4% of patients with PC survive five years after diagnosis (3).

Tumor metastasis is the dissemination and proliferation of cancer cells in an organ that is distinct from the primary site (4). Metastasis is also the most common cause of death in cancer patients, accounting for approximately 90% of cancer-related deaths (5). This is particularly true for pancreatic cancer, in which most cases are diagnosed with metastatic disease or develop distant metastases after surgical resection. Liver metastasis represents the first site of metastasis in more than 80% of metastatic pancreatic cancer (6). Lung metastasis, as the site of dissemination, is a relatively rare event and has been suggested to define a unique clinical subgroup of pancreatic cancer (6–10). For cases with recurrence after resection of PC with curative intent, previous studies reported that recurrence in the lung ranged from 9.8% to 16.1% of all cases (6, 10). Previous studies have reported a favorable prognosis for pancreatic cancer with lung metastases. Oweira et al. reported that pancreatic cancer patients with isolated lung metastases had better overall and cancer-specific survival than patients with isolated liver metastases (11). A previous study also suggested favorable survival in patients with isolated lung metastases recurrences after surgical resection of the primary tumor, which represents a favorable prognostic factor that is independent of the time to recurrence in pancreatic cancer (6). However, previous reports on pancreatic cancer with lung metastasis (PCLM) have primarily focused on case reports and single-center case series. Owing to the small sample size and low credibility of these studies, there is an apparent deficiency in the field’s understanding of the clinicopathological features and prognosis of patients with PCLM. To address these questions, we conducted a retrospective study using data from the Surveillance, Epidemiology, and End Results (SEER) database to explore the risk and prognostic factors of these patients.

## Methods and materials

### Participants

The SEER database is the largest publicly available cancer database. The SEER program collected patient information from various registries throughout the United States (U.S.), accounting for 28% of the U.S. population, including demographics, clinicopathological characteristics, treatment regimen, and survival. We used SEER*Stat 8.3.8 software to identify pancreatic cancer patients based on the database “Incidence-SEER 18 Regs Research Data (with additional treatment field) Nov 2018 Sub (1975-2016 varying)”. Patients with a diagnosis of primary pancreatic cancer were identified using histological types (International Classification of Diseases [ICD]-0-3: 8010, 8020, 8021, 8140, 8480, 8481, and 8500) and from the corresponding locations (Site recode ICD-O-3/World Health Organization [WHO] 2008: pancreas). We excluded patients with pancreatic cancer whose metastasis status and follow-up information were unknown/incomplete and those diagnosed with multiple primary cancers.

### Variables

Patients with adequate data on distant metastases could be included in the analysis of the incidence of PCLM and lung metastasis patterns among patients with pancreatic cancer. To be included in the analysis of risk and prognostic factors for pancreatic cancer with lung metastases, patients were required to have clear and complete information regarding age, sex, race, marital status, insurance status, histological subtype, pathological grade, TNM stage, lymph node metastases, distant metastases, and survival. The study endpoints for prognosis analysis were overall survival (OS) and pancreatic cancer-specific survival (PCSS). OS was defined as the time from initial diagnosis to death caused by any reason or the data of the last follow-up. PCSS was defined as the interval between the initial diagnosis and death due to pancreatic cancer.

### Statistical analysis

The demographic and clinicopathological characteristics of patients were presented using counts and percentages, and the differences in categorical and continuous variables were analyzed using the chi-squared test and Student’s t-test. The OS and CSS of patients with PCLM were described using Kaplan–Meier curves. Univariate and multivariable logistic regression analyses were used to determine the risk factors for lung metastasis in patients with PC. Univariate and multivariable Cox regression models were used to evaluate the association of each variable with prognosis and to identify independent prognostic factors for PCLM patients. In addition, we constructed nomograms based on independent risk factors and prognostic factors to predict the risk and survival of PCLM. The accuracy of the established nomograms was evaluated using the C-index and receiver operating characteristic (ROC) analysis, and the discrimination of the nomograms was confirmed using calibration plots. Decision curve analysis (DCA) was used to evaluate the clinical utility of the established predictive models (12). All analyses were performed using MedCal software () and R statistical software (version 4.1. http://www.R-project.org/) with the packages “stringr”, “ggplot2”, “tableone”, “surviminer”, “survival”, “ggDCA”, “rmda”, “pROC, etc. Statistical significance was set at P<0.05.

## Results

### Patients’ clinical characteristics

A total of 50,959 patients with PC between 2010 and 2016 were identified in the SEER database. To further describe the clinicopathological characteristics of PCLM, patients without information on the aforementioned variables were excluded and 11,287 cases were finally included in the present study. Of the 11,287 patients with PC in the present cohort, 601 (5.3%) had lung metastases when diagnosed with PC.

As shown in **Table 1**, there were significant differences in insurance status, primary site, histological subtype, pathological grade, T stage, and liver/bone/brain metastases between those with PCLM and those without PCLM. Patients with PCLM tended to be uninsured and had a histological diagnosis of adenocarcinoma (75.4% vs. 63.0%). Patients with PCLM were also more likely to be diagnosed with pathological grade III/IV (52.3% vs. 42.6%), T4 (28.3% vs. 16.4%), liver metastases (57.1% vs. 17.2%), bone metastases (12.1% vs. 1.1%), and brain metastases (1.2% vs. 0.1%). Among the primary sites, PC was less likely to occur in the pancreatic head (37.3% vs. 66.0%). Regarding treatment regimens, patients with PCLM were less likely to undergo surgery (6.8% vs. 59.3%), radiation (9.3% vs. 25.9%), and chemotherapy (56.1% vs. 67.5%) (**Table 1**).

**Table 1:** Baseline clinicopathological features and treatment regimen of PCLM.

Feature	Without lung metastases (N=10686)	With lung metastases (N=601)	p-Value
**Age**			
<60 years	2784 (26.1%)	150 (25.0%)	0.51
60-74 years	5227 (48.9%)	288 (47.9%)	
≥75 years	2675 (25.0%)	163 (27.1%)	
**Sex**			
Female	5193 (48.6%)	297 (49.4%)	0.70
Male	5493 (51.4%)	304 (50.6%)	
**Race**			
White	8490 (79.4%)	468 (77.9%)	0.40
Black	1264 (11.8%)	71 (11.8%)	
Other (American Indian/AK Native, Asian/Pacific Islander)	932 (8.7%)	62 (10.3%)	
**Marital status**			
Married	6285 (58.8%)	331 (55.1%)	0.15
Unmarried	1433 (13.4%)	93 (15.5%)	
Other	2968 (27.8%)	177 (29.5%)	
**Insurance**			
Insured	9085 (85.0%)	485 (80.7%)	0.02
Uninsured	296 (2.8%)	23 (3.8%)	
Other	1305 (12.2%)	93 (15.5%)	
**Site**			
Head of pancreas	7056 (66.0%)	224 (37.3%)	<0.01
Body of pancreas	1149 (10.8%)	107 (17.8%)	
Tail of pancreas	1121 (10.5%)	134 (22.3%)	
Overlapping lesion of pancreas	673 (6.3%)	64 (10.6%)	
Other	687 (6.4%)	72 (12.0%)	
**Histology**			
Adenocarcinoma	6733 (63.0%)	453 (75.4%)	<0.01
Infiltrating duct carcinoma	3289 (30.8%)	73 (12.1%)	
Other	664 (6.2%)	75 (12.5%)	
**Pathological grade**			
Grade I	1229 (11.5%)	56 (9.3%)	<0.01
Grade II	4911 (46.0%)	231 (38.4%)	
Grade III	4334 (40.6%)	294 (48.9%)	
Grade IV	212 (2.0%)	20 (3.3%)	
**T**			
T0	13 (0.1%)	8 (1.3%)	<0.01
T1	443 (4.1%)	21 (3.5%)	
T2	1675 (15.7%)	163 (27.1%)	
T3	6804 (63.7%)	239 (39.8%)	
T4	1751 (16.4%)	170 (28.3%)	
**N**			
N0	4931 (46.1%)	281 (46.8%)	0.76
N1	5755 (53.9%)	320 (53.2%)	
**Liver**			
No	8850 (82.8%)	258 (42.9%)	<0.01
Yes	1836 (17.2%)	343 (57.1%)	
**Bone**			
No	10573 (98.9%)	528 (87.9%)	<0.01
Yes	113 (1.1%)	73 (12.1%)	
**Brain**			
No	10679 (99.9%)	594 (98.8%)	<0.01
Yes	7 (0.1%)	7 (1.2%)	
**Radiation**			
None	7917 (74.1%)	545 (90.7%)	<0.01
Yes	2769 (25.9%)	56 (9.3%)	
**Surgery**			
None	4345 (40.7%)	560 (93.2%)	<0.01
Yes	6341 (59.3%)	41 (6.8%)	
**Chemotherapy**			
No/Unknown	3476 (32.5%)	264 (43.9%)	<0.01
Yes	7210 (67.5%)	337 (56.1%)	

The bold values mean P<0.05.

According to the lung metastases pattern, PCLM patients were divided into two cohorts: only lung metastases (N=203) and not-only metastases (n=398). The clinicopathological features and treatment regimens between these two cohorts were compared (**Supplementary Table 1**). PCLM Patients with only lung metastases tended to be female and had a pathological grade III/IV. Regarding treatment regimens, PCLM patients with only lung metastases were less likely to undergo surgery (0.5% vs. 11.1%), radiation (5.2% vs. 11.6%), and more likely to receive chemotherapy (63.1% vs. 52.5%).

### Risk factors for PCLM

**Figure 1:**
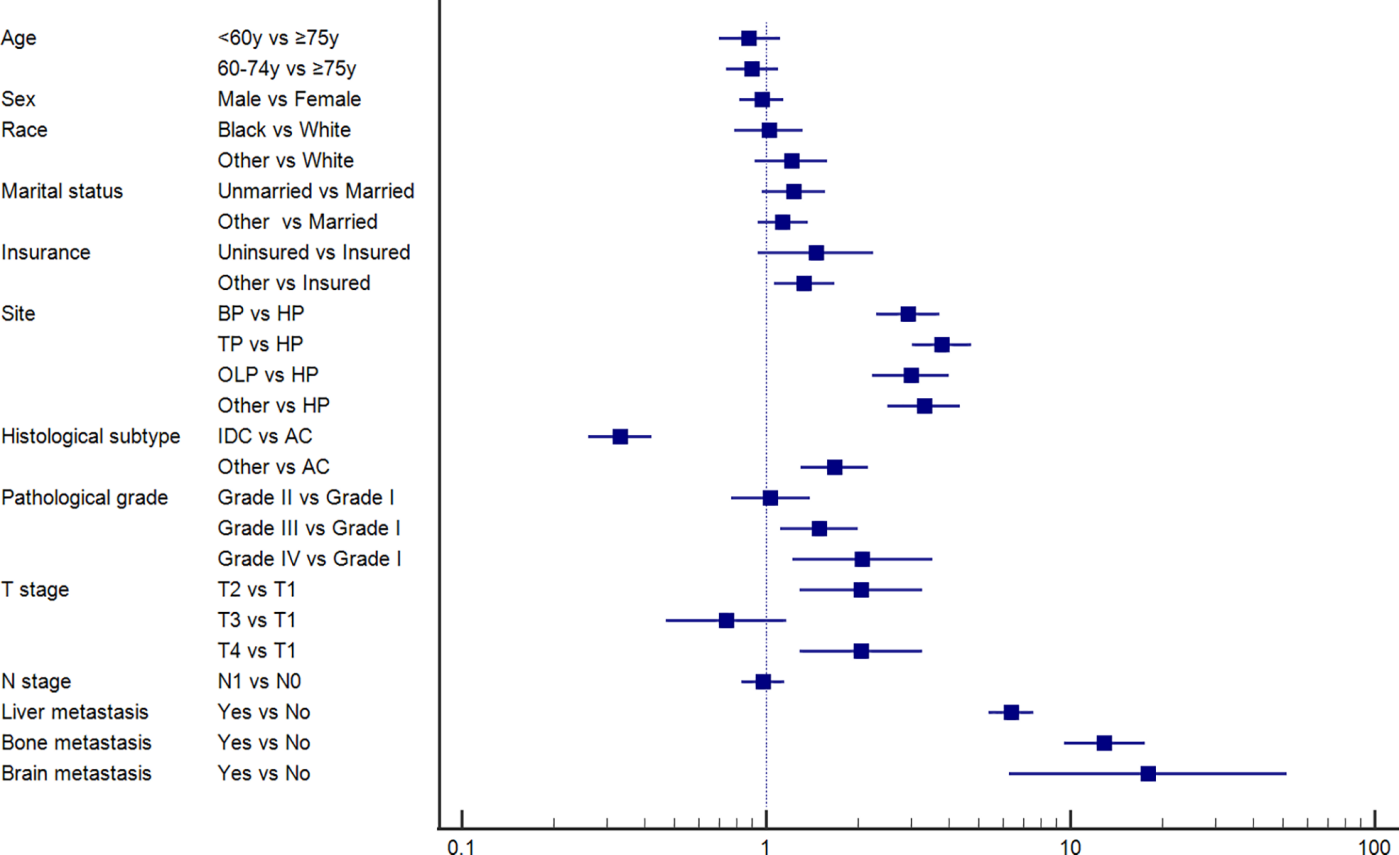
Forest plot showing the potential risk factor for lung metastasis among pancreatic cancer patients based on the logistic regression analysis.

Furthermore, we conducted univariate and multivariable logistic regression analyses to identify potential predictive factors for lung metastases among patients with PC. As shown in **Figure 1**, the forest plots showed that insurance status, primary site, histological subtype, pathological grade, T stage, and brain, bone, and liver metastases were associated with the development of lung metastasis (P<0.05). Further multivariable analysis demonstrated that primary site, histological subtype, and brain, bone, and liver metastases were independent risk factors for lung metastases among PC patients.

### Establishment of diagnostic nomogram for PCLM

**Figure 2:**
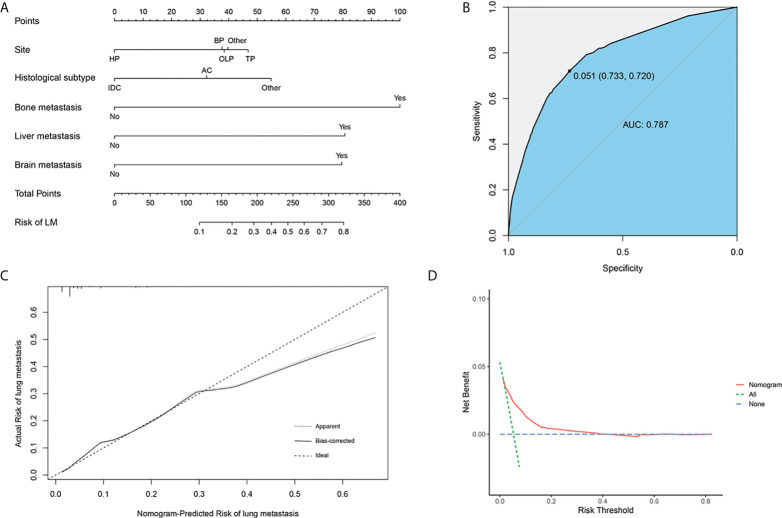
Nomogram to estimate the risk of lung metastasis in patients with pancreatic cancer **(A)**. The area under ROC curve was utilized to judge the advantages and disadvantages of nomogram **(B)**. Calibration plot for the diagnostic nomogram. The diagonal 45-degree line indicates perfect prediction **(C)**. Decision curve analysis for the diagnostic nomogram **(D)**. HP, head of pancreas; BP, body of pancreas; OLP, overlapping lesion of pancreas; TP, tail of pancreas; AC, Adenocarcinoma; IDC, Infiltrating duct carcinoma.

Based on the above independent predictors obtained from multivariable logistic analysis, we constructed a risk prediction nomogram model for developing lung metastases among patients with PC, and the c-index was 0.79 (95% CI 0.73-0.85) (**Figure 2A**). Bone metastases contributed the most to lung metastases development, followed by liver and brain metastases. ROC analysis revealed that the AUC value of the nomogram reached 0.787 (95% CI 0.763-0.814), indicating that this model has excellent discriminant ability (**Figure 2B**). The calibration curve showed that the observed results were highly consistent with the predicted results (**Figure 2C**). In addition, DCA showed that the nomogram model was effective in clinical practice (**Figure 2D**).

**Figure 3:**
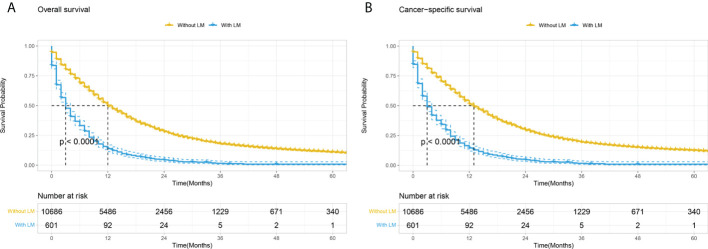
Kaplan–Meier curves comparing OS **(A)** and CSS **(B)** stratified by the absence or presence of lung metastases among PC patients. LM, Lung metastasis.

### Prognostic factors for PCLM

Patients with PCLM had significantly shorter OS and CSS than those without lung metastasis (mOS: 3 months vs. 12 months; mCSS: 3 months vs. 13 months; **Figure 3**). The 6-month, 12-month, 24-month OS rates for patients with PCLM were 33.2%, 13.9%, and 4.4%, respectively. The 1-year, 2-year, 5-year CSS rates were 34.2, 14.5, and 4.6%, respectively. Univariate and multivariable Cox regression analyses were used to identify potential variables that could influence survival among patients with PCLM The results demonstrated that primary site, liver metastases, surgery, and chemotherapy were independent prognostic factors for both OS and CSS among these patients, while bone metastases were independent prognostic factors for CSS (**Table 2**).

**Table 2:** Independent Prognostic factors for OS and CSS in PCLM by multivariable Cox analysis.

Characteristics	Multivariate Cox for OS		Multivariate Cox for CSS	
	HR (95%CI)	p-Value	HR (95%CI)	p-Value
**Age**				
<60 years	Reference		Reference	
60-74 years	1.00 (0.80-1.24)	0.97	1.08 (0.84-1.40)	0.55
≥75 years	1.12 (0.87-1.45)	0.38	0.97 (0.78-1.21)	0.79
**Sex**				
Female	Reference		Reference	
Male	0.94 (0.78-1.13)	0.53	0.93 (0.78-1.13)	0.48
**Race**				
White	Reference		Reference	
Black	1.15 (0.88-1.50)	0.30	1.28 (0.97-1.69)	0.08
Other	0.89 (0.72-1.10)	0.27	0.94 (0.70-1.26)	0.66
**Marital status**				
Married	Reference		Reference	
Unmarried	1.15 (0.88-1.50)	0.30	1.11 (0.85-1.45)	0.46
Other	0.89 (0.72-1.10)	0.27	0.89 (0.72-1.10)	0.27
**Insurance**				
Insured	Reference		Reference	
Uninsured	0.94 (0.60-1.47)	0.78	0.94 (0.73-1.22)	0.64
Other	0.95 (0.74-1.23)	0.71	0.96 (0.61-1.50)	0.85
**Site**				
Head of pancreas	Reference		Reference	
Body of pancreas	0.97 (0.76-1.25)	0.83	0.98 (0.76-1.27)	0.90
Tail of pancreas	**1.27 (1.01-1.60)**	**0.04**	**1.29 (1.02-1.64)**	**0.03**
Overlapping lesion of pancreas	1.28 (0.95-1.73)	0.10	1.25 (0.93-1.70)	0.14
Other	0.89 (0.66-1.19)	0.42	0.86 (0.63-1.16)	0.32
**Histology**				
Adenocarcinoma	Reference		Reference	
Infiltrating duct carcinoma	0.86 (0.65-1.14)	0.30	0.86 (0.65-1.15)	0.31
Other	1.01 (0.78-1.32)	0.93	1.00 (0.76-1.31)	0.99
**Grade**				
Grade I	Reference		Reference	
Grade II	1.02 (0.75-1.40)	0.90	1.03 (0.75-1.41)	0.88
Grade III	1.16 (0.85-1.59)	0.35	1.14 (0.83-1.57)	0.41
Grade IV	1.55 (0.89-2.67)	0.12	1.59 (0.92-2.75)	0.10
**T**				
T1	Reference		Reference	
T2	1.25 (0.78-2.01)	0.35	1.25 (0.78-2.00)	0.36
T3	0.80 (0.50-1.28)	0.35	0.79 (0.49-1.26)	0.31
T4	1.14 (0.71-1.84)	0.58	1.11 (0.69-1.78)	0.68
**N**				
N0	Reference		Reference	
N1	1.18 (0.98-1.42)	0.07	1.18 (0.98-1.42)	0.08
**Liver**				
No	Reference		Reference	
Yes	**1.51 (1.24-1.82)**	**<0.01**	**1.52 (1.25-1.84)**	**<0.01**
**Bone**				
No	Reference		Reference	
Yes	**1.36 (1.03-1.79)**	**0.03**	1.31 (0.99-1.73)	0.06
**Brain**				
No	Reference		Reference	
Yes	1.21 (0.54-2.69)	0.64	1.08 (0.46-2.53)	0.87
**Radiation**				
None/Unknown	Reference		Reference	
Yes	0.91 (0.67-1.23)	0.53	0.93 (0.68-1.26)	0.63
**Surgery**				
None	Reference		Reference	
Yes	**0.56 (0.39-0.83)**	**<0.01**	**0.54 (0.37-0.80)**	**<0.01**
**Chemotherapy**				
No/Unknown	Reference		Reference	
Yes	**0.27 (0.22-0.34)**	**<0.01**	**0.27 (0.22-0.33)**	**<0.01**

The bold values mean P<0.05.

In addition, the survival analysis showed that patients with only lung metastases had significantly better OS and CSS than those with not-only lung metastases (P<0.01 for both, **Supplementary Figure 1**). The analysis also showed that chemotherapy could significantly prolong both OS and CSS of patients with only lung metastases (P<0.01 for both), but radiation could not improve the prognosis of these patients (**Supplementary Figure 2**).

### Establishment of prognostic nomogram for PCLM

**Figure 4:**
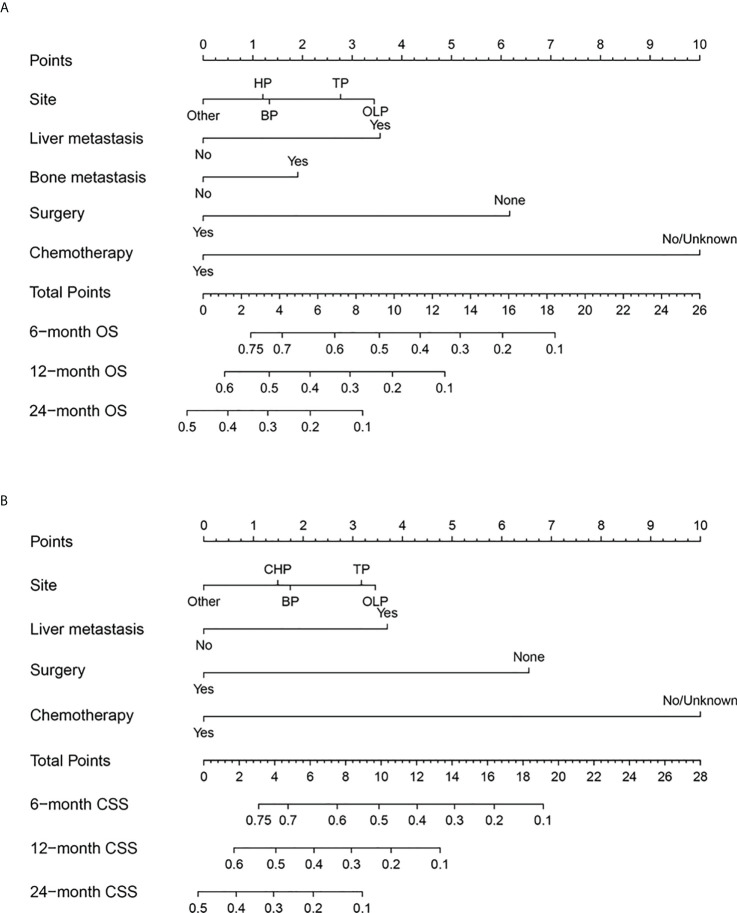
Prognostic nomogram to predict OS **(A)** and CSS **(B)** for the 6-, 12-, and 24-month survival for PCLM patients.

**Figure 5:**
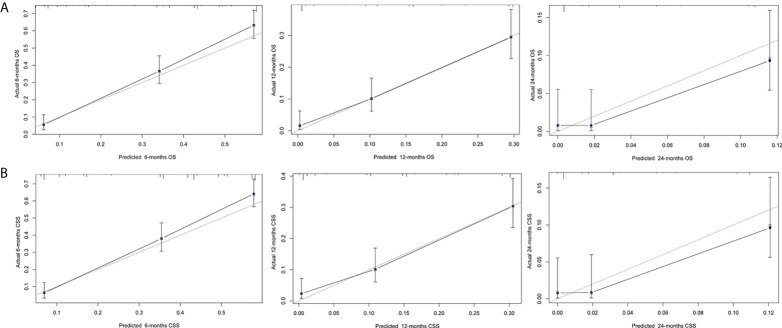
Calibration plot (**A**: OS, **B**: CSS) for prognostic nomograms.

**Figure 6:**
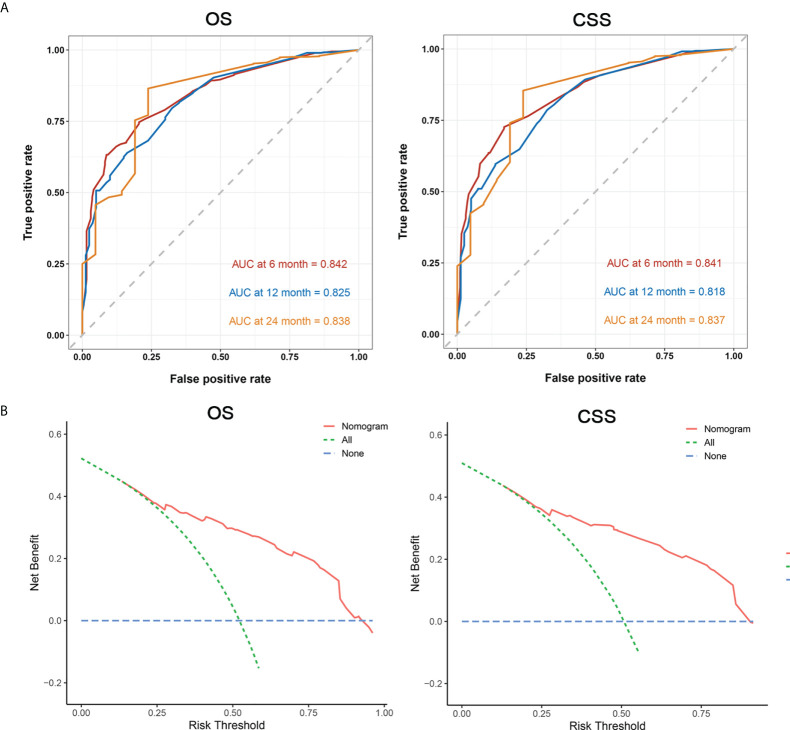
Area under ROC curve (**A**, left: OS, right: CSS), and DCA (**B**, left: OS, right: CSS) for prognostic nomograms.

Next, we established a prognostic nomogram to predict the OS and CSS of patients with PCLM using these independent prognostic factors from the multivariable Cox regression analysis. As shown in **Figure 4**, chemotherapy contributed the most to the prediction of OS and CSS, followed by surgical resection of the primary tumor. The C-indices for OS and CSS prediction were 0.76 (95% CI 0.74-0.78) and 0.76 (95% CI 0.74-0.78), respectively. Calibration plots for the established nomograms showed that the predicted 6-, 12-, and 24-month OS and CSS probabilities were almost identical to actual observations (**Figure 5**). ROC analysis of the OS-specific nomogram revealed that the AUC of 6-month, 12-month, 24-month OS reached 0.842, 0.825, and 0.838, respectively, and 0.841, 0.818, and 0.837, respectively, for CSS prediction (**Figure 6**). DCA has been widely used to assess the clinical value of established OS-and CSS-specific nomograms. As illustrated in **Figure 6**, the nomogram demonstrated a significant positive net benefit from the risk of death, suggesting its practical, clinical, and real-world value in predicting OS and CSS among patients with PCLM.

## Discussion

Although the lungs are the most common extrahepatic site of distant metastasis among PC patients, most previous studies on PCLM are case reports or single-center case series. To date, there have been few reports regarding the risk and prognostic factors of PCLM. To the best of our knowledge, our research is the first SEER-based comprehensive retrospective study to focus on the establishment of nomogram models to predict the risk and prognosis of lung metastases among patients with PC. These models are helpful for physicians to better manage patients with PC in clinical practice.

In our study, the risk factors for developing lung metastases among patients with PC included primary site, histological subtype, brain metastasis, liver metastasis, and bone metastasis. We established a diagnostic nomogram for predicting PCLM, calibration plot, ROC curve, and DCA, which revealed that the nomogram possessed considerable predictive power. Thus, physicians should pay close attention to these risk factors for PC patients and allow high-risk patients to receive earlier lung scans to determine the presence of lung metastases.

A previous study using 13,233 patients with stage IV pancreatic ductal adenocarcinoma from the SEER database found that patients with lung metastases had better survival than those with liver metastases (11). Recently, a pooled analysis of three large randomized trials (CONKO-001, CONKO-005, and CONKO-006) demonstrated isolated pulmonary recurrence as an independent favorable prognostic factor in patients with PDAC who experienced relapse after postoperative adjuvant chemotherapy. However, it remains unclear which factors affect the survival of patients with PC. We conducted a multivariable Cox regression analysis for OS and CSS among patients with PCLM. Survival analysis showed that primary site, liver metastasis, use of chemotherapy, and surgery were significant predictors for both OS and CSS among patients with PCLM, and bone metastasis was only independently associated with OS. Similarly, we constructed OS-/CSS-specific nomograms using these prognostic factors, and physicians could effectively and accurately predict the prognosis and provide clinical guidance for patients with PCLM.

Similar to other digestive system malignancies, pancreatic cancer has a characteristic trend of preferential metastasis to the liver, followed by the lung; however, bone metastasis of PC is almost as rare as brain metastasis (13, 14). Interestingly, liver, bone, and brain metastases are risk factors for PCLM. As for prognosis prediction, although brain metastasis was a risk factor for pancreatic cancer with bone metastases (15), it had no significant impact on the survival of patients with PCLM. One possibility for this is the low proportion of brain metastases in the present cohort; only 14 out of 601 patients with metastatic PC developed brain metastases at initial diagnosis. In addition, bone and liver metastases were independently associated with the prognosis of PLCM, suggesting that physicians should take timely and effective measures to prevent bone or liver metastasis following lung metastases. Furthermore, clinicians should pay close attention towards screening bone and liver metastases in order to accurately predict the prognosis.

Adenocarcinoma was often considered to be the most prevalent histological subtype of pancreatic cancer, followed by infiltrating duct carcinoma, with 63.8% and 29.8% of cases diagnosed with adenocarcinoma and IDC in our cohort, respectively. Previous studies have reported that pancreatic adenocarcinoma exhibits the lowest overall survival and worse prognosis among patients with PC, and adenocarcinoma is also an independent prognostic factor for overall survival among patients with PC with bone metastases. In the present study, we did not observe a significant correlation between the pathological subtype and OS or CSS of PCLM patients with PCLM. However, PC patients with adenocarcinoma are more likely to develop lung metastases than those with IDC. This is inconsistent with the study performed by Zhang et al., which showed that patients with adenocarcinoma have a low risk of bone metastases (15). A previous study demonstrated that genetic alterations present in metastatic lesions reflect the mutational landscape in the founder clone and might determine the metastatic pattern of PC; the metastatic pattern of PC could therefore indicate distinct clinical and genetic subgroups (4). Thus, we speculate that intrinsic genetic differences may contribute to the difference in risk factors and prognostic factors between patients with PCLM and bone metastases.

In addition, our data showed that the primary site could also affect the risk and prognosis of patients with PCLM. Pancreatic cancer occurring in the head of the pancreas was less likely to develop lung metastasis and had a relatively better survival. In addition, our data also showed that patients with lesion in tail of pancreas have more likely to develop distant organ metastasis including bone, brain, liver. This is not specific to the lung metastases. These results are inconsistent with those of the previous studies. Kovac et al. conducted a retrospective study of 90 patients with recurrent pancreatic cancer after surgical resection and demonstrated that patients with pancreatic head tumors had a lower incidence of metastatic disease than those with pancreatic body and/or tail carcinoma (16). Currently, chemotherapy is the main modality for first-line treatment of metastatic pancreatic cancer (17). Our data also showed that chemotherapy could significantly prolong OS and CSS of PCLM patients. As for surgery, pulmonary metastasectomy remains a non-standard and individual treatment approach, and increasing interest in pulmonary metastasectomy has recently increased, with numerous case reports and case series. Kruger reported that patients with recurrent PCLM who underwent surgical metastasectomy for lung metastases appeared to have improved outcomes compared to patients who did not undergo surgery (6). Kurahara et al. reported that this clinical benefit was limited to single lung metastasis and was significantly associated with a lower FDG-PET SUVmax of the primary pancreatic tumor (18). Groot VP et al. reported that pulmonary metastasectomy could be considered in highly selected patients including good performance status, and recurrence occurs after a recurrence-free interval of more than 16 months, etc. (19). Consistent with these results, our data also reported the clinical benefits of surgical resection for PCLM. However, the appropriate selection of patients with a favorable tumor biology for pulmonary metastasectomy remains unclear and requires further exploration.

There are several potential limitations to this study. First, the ROC curve, calibration curve, and DCA were used to evaluate the accuracy of the established nomogram. The nomogram is based only on retrospective studies, without any validation cohort, which limits the accuracy and reliability of the present results. Second, other factors that potentially affect survival, such as specific surgical procedure, chemotherapy regimen, performance status, comorbidities, and resection margin status, were not available in the SEER database, which may have led to a bias in the analysis. Besides, the distant metastases pattern recorded in the SEER database only included lung/liver/bone/brain (after 2010 years), as well as distant lymph node (after 2016 years). The patients with other organ metastases were not recorded in the SEER database. This may limit the accuracy and reliability of clinicopathological and prognosis of PCLM patients with isolated lung metastases. Despite these limitations, we believe that the results of this study will provide clinicians with further insight into this rare tumor.

In conclusion, we first identified the risk and prognostic factors of PCLM using data from the SEER database and established two predictive nomograms. These nomograms can help clinicians effectively identify high-risk patients and predict clinical outcomes. These results are essential for the disease management of this type of pancreatic cancer, as well as for future prospective studies examining this disease.

## Data availability statement

The raw data supporting the conclusions of this article will be made available by the authors, without undue reservation.

## Author contributions

Conception and design: Z-XY, J-HT, and X-FX; Provision of study materials or patients: Z-XY and J-HT; Collection and assembly of data: Z-XY, J-HT, BZ, and YH; Data analysis and interpretation: Z-XY, J-HT, and Y-LL; Manuscript writing: All authors. All authors contributed to the article and approved the submitted version.

## Acknowledgments

We thank all the staff who participated in this study, especially the SEER database for kindly providing valuable data resources (http://seer.cancer.gov/).

## Conflict of interest

The authors declare that the research was conducted in the absence of any commercial or financial relationships that could be construed as a potential conflict of interest.

## Publisher’s note

All claims expressed in this article are solely those of the authors and do not necessarily represent those of their affiliated organizations, or those of the publisher, the editors and the reviewers. Any product that may be evaluated in this article, or claim that may be made by its manufacturer, is not guaranteed or endorsed by the publisher.
