# Supplementary figures and images for: Risk factors and survival prediction of pancreatic cancer with lung metastases: A population-based study
Source: Front Oncol. 2022 Sep 21;12:952531. doi: 10.3389/fonc.2022.952531 (PMC9533144; doi:10.3389/fonc.2022.952531)

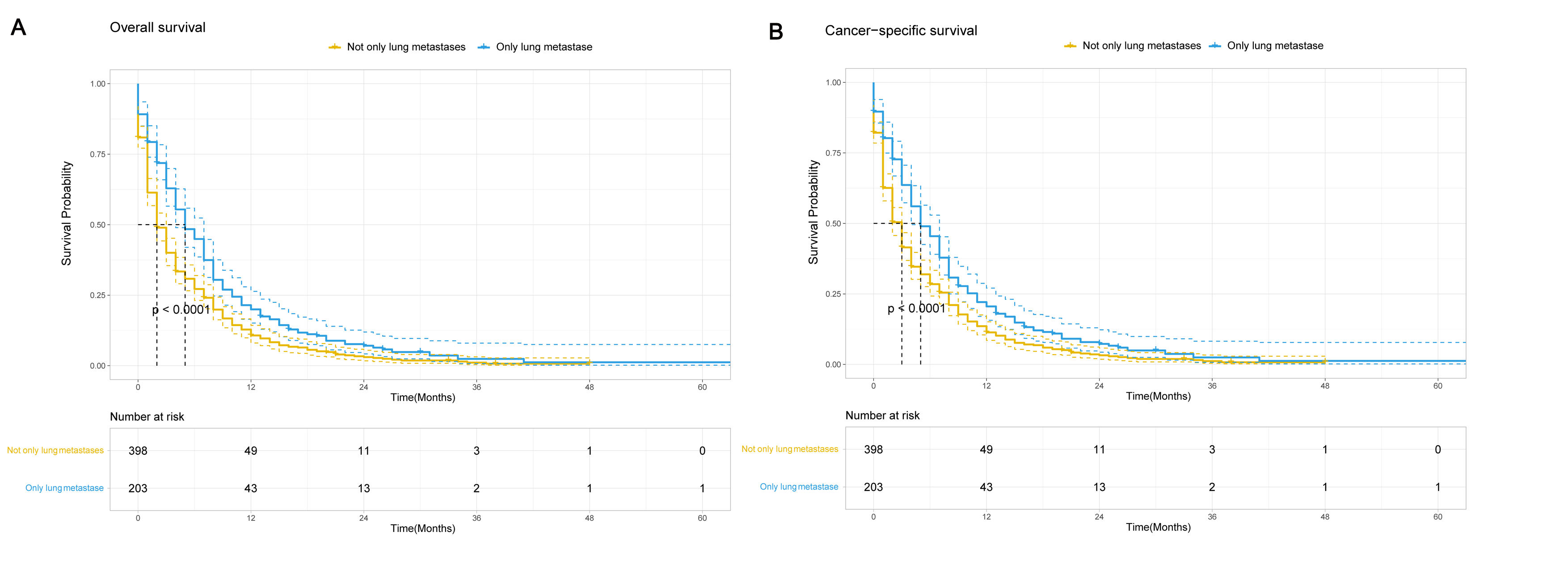

Supplement: Supplementary file 1 [file Image_1.tif]

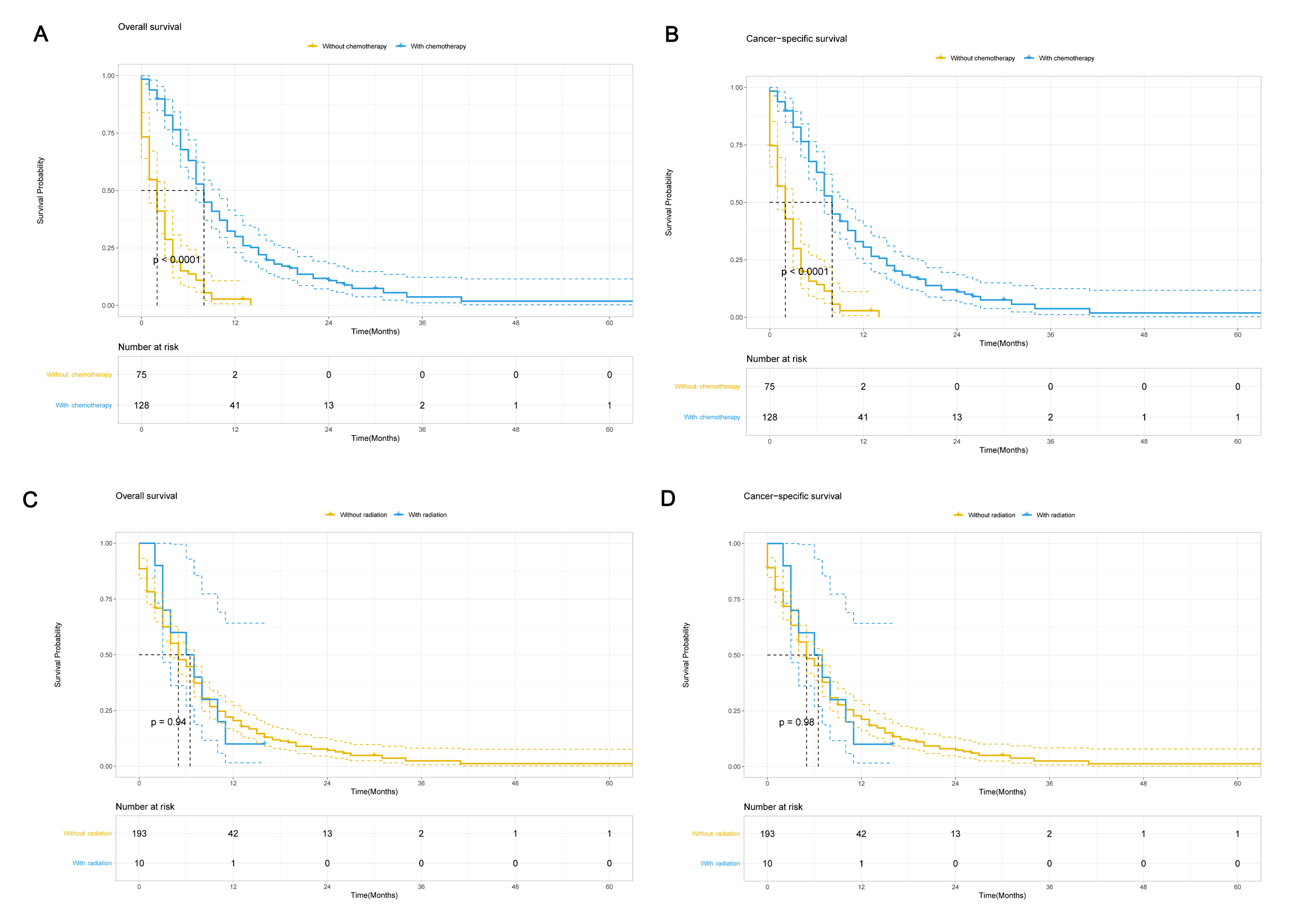

Supplement: Supplementary file 2 [file Image_2.tif]
