# Supplementary material for: Risk factors and survival prediction of pancreatic cancer with lung metastases: A population-based study
Source: Front Oncol. 2022 Sep 21;12:952531. doi: 10.3389/fonc.2022.952531 (PMC9533144; doi:10.3389/fonc.2022.952531)
Supplement: Supplementary file 3 [file Table_1.docx]

**Supplementary Table 1:** Baseline clinicopathological features and treatment regimen of only lung metastases and not-only lung metastases

| Feature | Only lung metastases (N=203) | Not-Only lung metastases (N=398) | p-Value |
| --- | --- | --- | --- |
| **Age** |  |  |  |
| <60 years | 44 | 106 | 0.04 |
| 60-74 years | 68 | 95 |  |
| ≥75 years | 91 | 197 |  |
| **Sex** |  |  |  |
| Female | 119 | 178 | <0.01 |
| Male | 84 | 220 |  |
| **Race** |  |  |  |
| White | 159 | 309 | 0.65 |
| Black | 21 | 50 |  |
| Other (American Indian/AK Native, Asian/Pacific Islander) | 23 | 39 |  |
| **Marital status** |  |  |  |
| Married | 110 | 221 | 0.10 |
| Unmarried | 21 | 72 |  |
| Other | 72 | 105 |  |
| **Insurance** |  |  |  |
| Insured | 173 | 312 | 0.08 |
| Uninsured | 4 | 19 |  |
| Other | 26 | 67 |  |
| **Site** |  |  |  |
| Head of pancreas | 86 | 138 | 0.03 |
| Body of pancreas | 43 | 64 |  |
| Tail of pancreas | 34 | 100 |  |
| Overlapping lesion of pancreas | 16 | 48 |  |
| Other | 24 | 48 |  |
| **Histology** |  |  |  |
| Adenocarcinoma | 152 | 301 | 0.98 |
| Infiltrating duct carcinoma | 25 | 48 |  |
| Other | 26 | 49 |  |
| **Pathological grade** |  |  |  |
| Grade I | 35 | 21 | <0.01 |
| Grade II | 84 | 147 |  |
| Grade III | 79 | 215 |  |
| Grade IV | 5 | 15 |  |
| **T** |  |  |  |
| T0 | 1 | 7 | 0.13 |
| T1 | 9 | 12 |  |
| T2 | 65 | 98 |  |
| T3 | 70 | 169 |  |
| T4 | 58 | 112 |  |
| **N** |  |  |  |
| N0 | 105 | 176 | 0.08 |
| N1 | 98 | 222 |  |
| **Radiation** |  |  |  |
| None | 193 | 352 | 0.01 |
| Yes | 10 | 46 |  |
| **Surgery** |  |  |  |
| None | 202 | 358 | <0.01 |
| Yes | 1 | 40 |  |
| **Chemotherapy** |  |  |  |
| No/Unknown | 75 | 189 | 0.02 |
| Yes | 128 | 209 |  |
